# Endotherapic treatment to control *Toumeyella parvicornis* Cockerell infestations on *Pinus pinea* L

**DOI:** 10.1002/ps.6876

**Published:** 2022-04-04

**Authors:** Nicolò Di Sora, Luca Rossini, Mario Contarini, Enrico Chiarot, Stefano Speranza

**Affiliations:** ^1^ Dipartimento di Scienze Agrarie e Forestali Università degli Studi della Tuscia Viterbo Italy

**Keywords:** invasive species, pine tortoise scale, IPM, stone pine, insecticide persistence

## Abstract

**BACKGROUND:**

The pine tortoise scale, *Toumeyella parvicornis* (Cockerell, 1897), is a damaging insect pest native to North America. Its accidental introduction into Europe, where it was first reported in central‐southern Italy, is leading to severe infestations among stone pine trees, *Pinus pinea* L. causing severe infestations and generating a major risk to the health and safety of the citizens as well. This preliminary study aimed at finding an effective low‐impact control strategy against *Toumeyella parvicornis*. We evaluated the effect of endotherapic abamectin injected into infested stone pines in the Parco Archeologico di Ostia Antica (Rome).

**RESULTS:**

Results showed that endotherapic abamectin significantly reduced the pine tortoise adult female populations and had a persistence into plants of approximately 60 days. The first trace of abamectin on the plant's crown was detected 1 month after the treatment. Moreover, the survey highlighted a higher presence of the pest on the twigs of the plants than on needles.

**CONCLUSIONS:**

These findings offer an important tool in fighting the damaging activity of this phytophagous, especially in an urban context where interventions with treatments are strictly regulated by national laws. Endotherapy, in fact, would reduce the dispersion of active ingredients by drift, an aspect that could represent a valid alternative to manage plants in public areas. Given the actual lack of scientific information about other control solutions, abamectin endotherapic treatments would be the more effective strategy currently applicable. © 2022 The Authors. *Pest Management Science* published by John Wiley & Sons Ltd on behalf of Society of Chemical Industry.

## Graphical abstract

Abamectin insecticide through endotherapy treatment, is effective to control *Toumeyella parvicornis* affecting *Pinus pinea*. The effect of the product starts 45 days after the treatment with a persistence of 60 days.

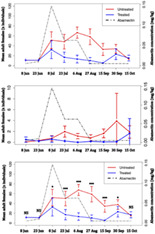

## 1 INTRODUCTION

*Toumeyella parvicornis* (Cockerell, 1897) (Hemiptera Coccidae), known as the pine tortoise scale, is a soft scale insect native to North America.^1^ Recently, it has been introduced into Italy,^2^ Puerto Rico^3^ and the Turks and Caicos Islands.^4^ In Italy, after its first report in Campania,^2^ *Toumeyella parvicornis* has spread and established in the Lazio Region.^5^, ^6^

The pest infests trees belonging to the genus *Pinus*,^7^ causing the plant dieback. The most infested species in Italy, *Pinus pinea* L., is not only a fundamental element of urban and suburban parks, but a widely perceived socio‐cultural symbol.^8^ The plant dieback caused by the insects’ feeding activity is related to symptoms such as reduction in shoot development, desiccation and yellowing of the needles and lack of vegetative renewal. The plant dieback becomes even more relevant in an urban environment, given that it increases the risk of accidental tree or branch falls towards the public. Besides safety, uncomfortable situations for citizens may arise from the great release of honeydew produced by the immature females, which endorses the growth of black moulds on pine twigs.^7^, ^9^

The biology of *Toumeyella parvicornis* has been partially explored: according to existing literature, the life cycles of both males and females are composed of four stages. The first stage is known as ‘crawler’ and is the only not‐static stage; the second and third stages are immature stages; and the fourth is the adult stage. The most prominent characteristic is the sexual dimorphism in adult stages, consisting in the presence of wings only in male adults.^7^, ^9^, ^10^

Although there is usually only one generation per year in North America,^11^ up to three overlapped generations per year have been recorded in Italy, likely favoured by warmer climate conditions.^7^

The entity of the infestations of *Toumeyella parvicornis* is leading scientists to investigate its biology,^7^ monitoring^10^ and control techniques.^12^ This work aims in particular to enrich the knowledge about the control of the species, reporting the results of the first year of an experiment conducted on infested stone pines in the Parco Archeologico di Ostia Antica (Rome, Italy). Specifically, it aims to evaluate the effect of endotherapic treatments on stone pines using the abamectin as active ingredient.

Abamectin‐based insecticides, in fact, have a large use in plant protection,^13^ showing strong results in efficacy (if applied through endotherapy) in controlling different pest species in urban environments. Among successful applications, it is worth mentioning the control of *Rhynchophorus ferrugineus* (Olivier) infesting palm trees,^14^ the control of the *Cameraria ohridella* (Deschka & Dimić) in horse‐chestnut trees,^15^ and the control of *Crisicoccus pini* (Kuwana)^16^ and of *Thaumetopoea pityocampa* (Denis & Schiffermüller)^17^ on pine trees.

The data obtained from the experiment were used for multiple objectives: (i) to assess the efficacy of the treatments by comparing the adult female population observed on a set of treated and untreated plants, and (ii) to estimate the persistence of abamectin in stone pines and the timing of its effectiveness in controlling *Toumeyella parvicornis*.

## 2 MATERIALS AND METHODS

### 2.1 Study area

Trials were conducted in the Parco Archeologico di Ostia Antica (Rome, Lazio, Italy, 41° 45′31.4″N, 12° 17′57.0″E). The stone pines involved in the study were distributed, at both the right and left sides along the main avenue of the park. A preliminary survey ascertained that all the plants were heavily infested by *Toumeyella parvicornis*, given the evident symptoms of black mould, desiccation and honeydew, typically related to the activity of the pest.

The experimental activities were carried out in 2021, from 24 May to 15 October.

### 2.2 Sampling design and treatment

**Figure 1:**
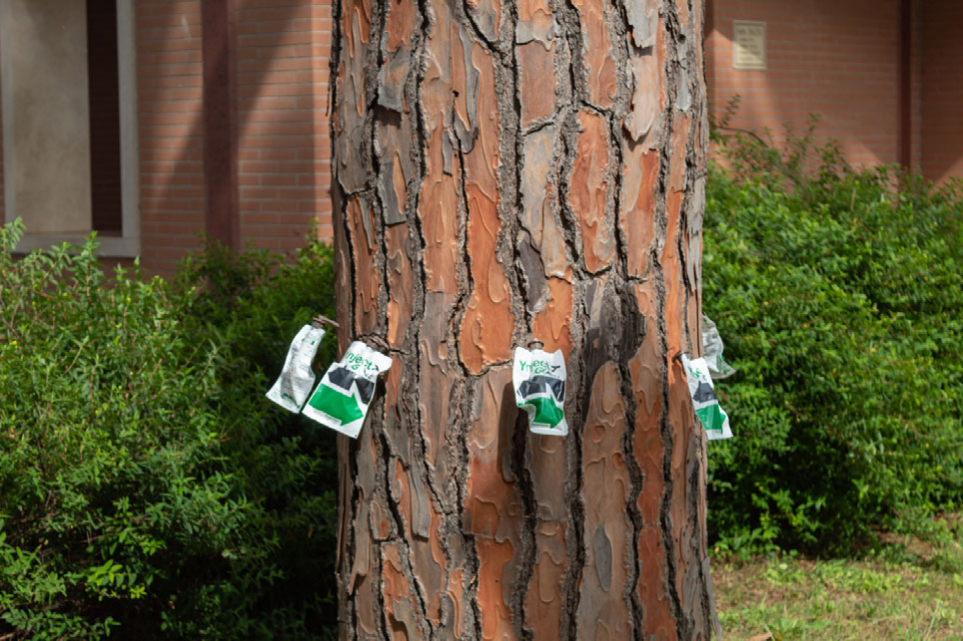
Endotherapic abamectin treatment on stone pine plants. Injection through the pressurized bags into the plant trunk.

Each of the two rows selected were composed of 20, 80‐years old stone pines, approximately 1.5 m in diameter and 20 m in height, spaced 6 m apart. Between the rows, five plants were randomly selected for endotherapic treatment, and the other five for untreated control. Endotherapic treatments on plants were carried out using the Ynject Go® technique provided by Fertinyect (Córdoba, Spain). This technique consists of a low‐pressure, high‐volume product application. A mixture composed of abamectin (6 mL) and co‐formulant (45 mL) was injected into the selected trees. Each injection was preceded by a perforation carried out at *c*. 1.5 m from the ground and at an angle of 30° using a drill bit of 5.5 mm diameter and 15 cm length. A total of six injections were carried out in each plant, maintaining a horizontal distance of 30 cm among the holes. The mixture was injected into the holes through a pressurized bag (Fig. 1), subsequently removed after complete emptying. A single treatment on the plants was carried out on 24 May 2021. It is also worth stating that we were not directly responsible for the treatments, since they were commissioned to a private company by the local authorities.

### 2.3 Samples collection

After the date of the endotherapic treatment, six 20 cm‐long twigs were randomly collected by reaching into the canopy of each treated and untreated plant with a basket crane and sampled fortnightly. After the cut, each twig was sealed into a plastic bag, brought to the laboratory and inspected within 24 h. Additionally, two other twigs were randomly collected by the treated plants and designated to multiresidue analysis.

### 2.4 Laboratory inspections

Samples were examined under a stereoscope, looking for adult females standing on twigs and on a selected number of needles (20 needles per twig). The collected individuals were identified following the illustrated keys^11^, ^18^ and counted with no distinction among the stages.

### 2.5 Multiresidue analysis

The multiresidue analysis was conducted on the ten additional twigs collected (twigs with needles), on each sampling date (Section 2.3). Samples were promptly brought to the laboratory and analysed using a liquid chromatography system, following the protocols available in Smith^19^ and widely used in contaminant and pesticide studies.^20^ The liquid chromatography system provided for two phases. The first phase, defined as mobile phase, provided for an aqueous buffer containing the twigs + needles mixture where, with a positive‐displacement pump, the flow rate was maintained constant. The buffer composition, instead, can be varied by taking fluids from an external tank. The second phase, defined as stationary phase, provided for the passage of the eluent by crosslinked agarose beads fixed in a cylindrical column where, through a detector, salt and protein concentrations can be measured by conductivity and ultraviolet light absorption (wavelength of 280 nm), respectively.^21^

### 2.6 Statistical analysis

The data analysis was divided into two steps and carried out using the R software (R Core Team 2018).

The first step of the analysis was directly related to the first objective of the present study, namely, understanding if the abamectin treatment is effective and where it is more likely to find adult females. It provided for a two‐factor analysis to assess: (i) the position, namely, which is the most likely part of the plant (between needles and twigs) to find adult females, and (ii) the efficacy of the endotherapic treatment. This analysis was carried out through a generalized linear model (GLM) with a negative binomial distribution and the Bonferroni adjusted as *post hoc* test (α=0.05), considering position and treatment as independent variables, and plants as a random variable. Calculations were performed through the glmr.nb() function within the R package lme4, the emmeans() and pairs() functions within the R packages multcompView and emmeans, and the cld() function within the R package multcomp.

The second step of the analysis was directly related to the second objective of the present study, which was to understand the timing of the treatment efficacy and its total coverage. It was carried out considering the summation of individuals collected on twigs and needles. A Mann–Whitney U test (wilcox.test() function within the R environment) was repeated on each sampling date to compare the population observed on treated and untreated plants. From this second part of the analysis, we aimed to estimate: (i) the time after injection required to observe significant differences between the adult female populations on treated and untreated plants, and (ii) how long abamectin in stone pines is effective in controlling adult female populations.

## 3 RESULTS

### 3.1 Population abundance and efficacy of abamectin

**Figure 2:**
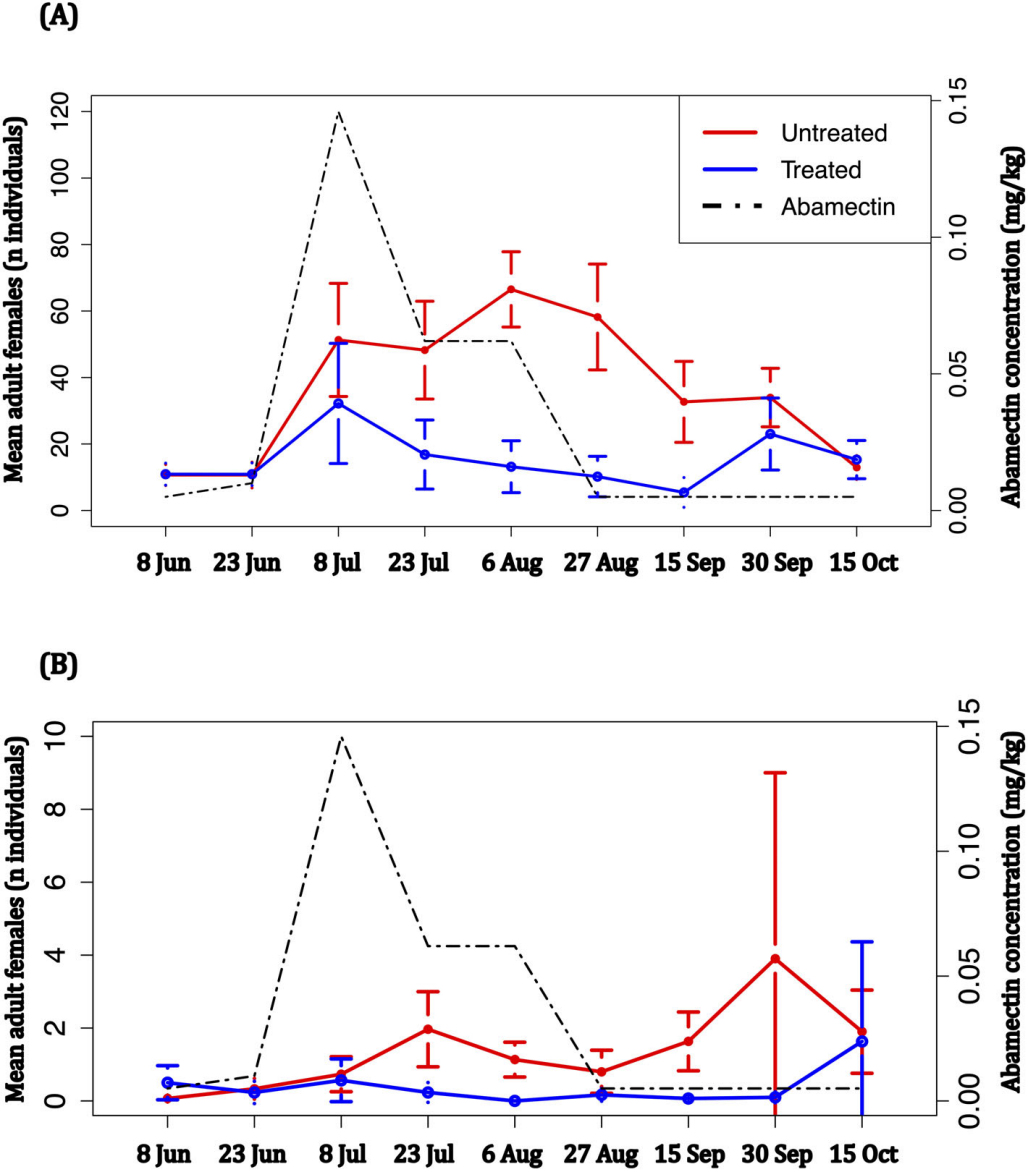
Average number of *Toumeyella parvicornis* adult females retrieved in each sampling date on treated (blue line) and untreated (red line) plants: (A) mean number of adult females collected on twigs, (B) mean number of adult females collected on needles. In both plots is reported the abamectin concentration measured on each sampling date (black dashed line). Error bars indicate the standard error of the mean (SEM).

From the first step of the analysis, it has been assessed, with statistical differences (GLM, *Z* = 31.759, *P* < 0.0001, NDF = 1075), that the number of adults on the needles was statistically lower than those observed on twigs (Fig. 2). Moreover, it has been ascertained a coherent statistical difference (GLM, *Z* = −3.506, *P* < 0.0001, Number of Degrees of Freedom (NDF) = 1075) between the populations observed on twigs of the treated and untreated plants (Fig. 2(A)) and the populations observed on needles of treated and untreated plants (Fig. 2(B)).

The results of the first analysis allowed us to consider the whole adult female population (twigs + needles) observed on treated and untreated plants to better explore their differences within each sampling date.

**Figure 3:**
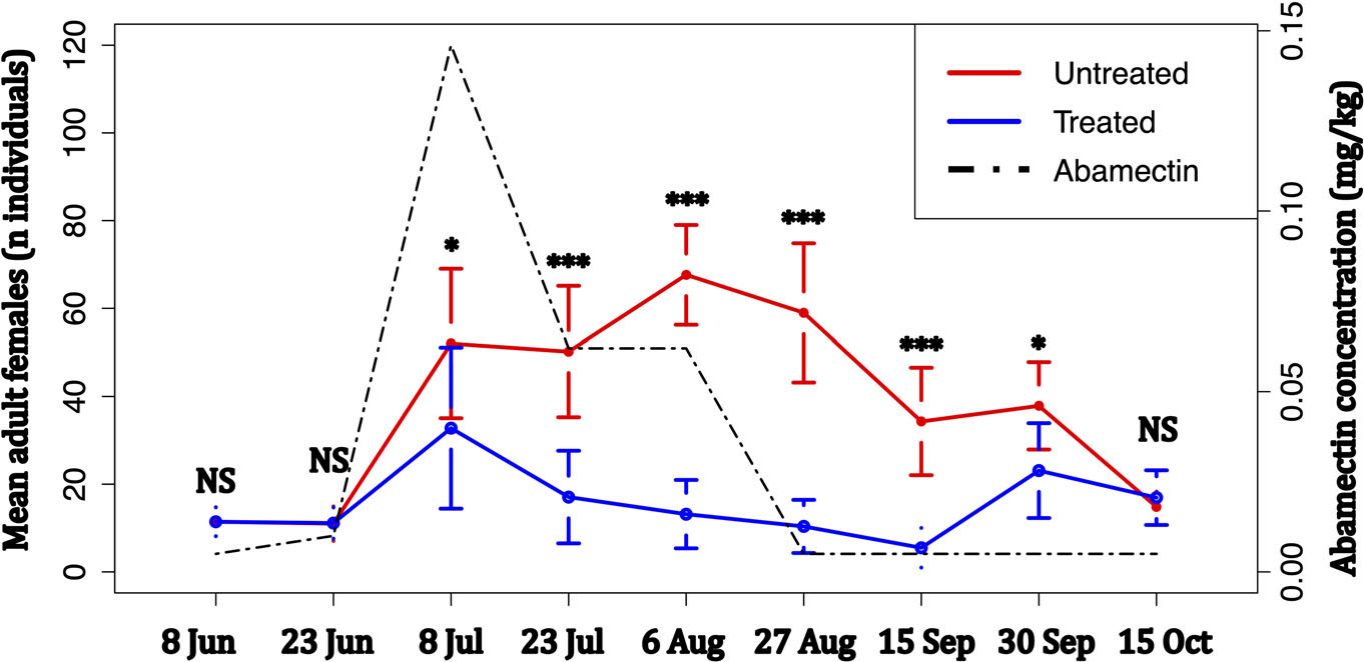
Average number of *Toumeyella parvicornis* adult females counted on each sampling date on treated and untreated plants (twigs + needles). Error bars indicate the standard error on the mean (SEM). Within each sampling date statistical differences, assessed through the Mann–Whitney U test, were reported with symbols *, **, ***, and NS indicating 0.05 ≤ *P* ≤ 0.01, 0.01 < *P* ≤ 0.001, *P* < 0.001 and *P* > 0.05, respectively.

Adult females on treated and untreated plants followed the same pattern until 8 July, as shown in Fig. 3. However, the Mann–Whitney U test showed that the populations were not statistically different on both the 8 June (MW, *W* = 431, *P* = 0.78) and 23 June (MW, *W* = 437.5, *P* = 0.85) samplings and statistically different on the 8 July sampling (MW, *W* = 610.5, *P* = 0.02). After the 8 July sampling the adult female populations’ trend on treated and untreated plants showed different patterns. On untreated plants, in fact, the number of individuals increased until 6 August, reaching a peak of 67 (mean of individuals per twigs), while *Toumeyella parvicornis* adult females counted on treated plants constantly decreased, reaching a minimum value on 15 September (Fig. 3). Statistical differences between the populations from 23 July to 15 September samplings were ascertained through the Mann–Whitney U test, where a *P* < 0.001 has been calculated for each date (MW, *W* = 688.5, *W* = 836.5, *W* = 813, *W* = 774.5, respectively).

A reduction of the differences between *Toumeyella parvicornis* adult female populations on treated and untreated plants was observed during the 30 September sampling. On this date, the reduction of the distance between the counts was coherent with a reduction of the statistical difference assessed with the Mann–Whitney U test (MW, *W* = 605, *P* = 0.02).

During the last sampling of 15 October, the adult females counted on treated and untreated plants showed similar values and no significant differences were reported by the Mann–Whitney U test (MW, *W* = 475.5, *P* = 0.71).

### 3.2 Multiresidue analysis

The endotherapic treatment was carried out on 24 May and the first trace of abamectin inside the plants was detected after 1 month (0.01 mg of abamectin per kilogram of plant tissue). The concentration reached a peak on 8 July (0.146 mg kg^−1^), 45 days after the injection, while after this date there was a decreasing trend shown by further monitoring. On 27 August, the amount of abamectin into pine plants maintained a concentration lower than 0.005 mg kg^−1^ (Figs 2 and 3) until the end of surveys. The multiresidue analysis conducted on treated plants showed that abamectin persists inside the pine tissues for around 50–60 days (Figs 2 and 3).

## 4 DISCUSSIONS

The results shown in this preliminary study are the first reporting relevant information about endotherapic abamectin as a potential control method of the newly introduced *Toumeyella parvicornis* on *P. pinea*.

The results indicate the strong potential of the treatment, given the significant decrease in population density observed in treated plants. Particularly, the population in treated plants slowed down its increase in correspondence with the maximum peak of abamectin concentration, maintaining low values for the whole duration of the survey. It is worth noting that the effect of the abamectin was coherently observed in both the twigs and needles populations, even though the species was significantly more present on twigs. The preference of *Toumeyella parvicornis* to stand more on twigs than on needles was previously observed by Orr^22^ and it is confirmed by our results. The reduction of the population density in treated plants represents a relevant starting point for future monitoring and control strategies for *Toumeyella parvicornis*, even though the persistence of abamectin inside the plant tissues is one of the most critical aspects to consider.

In the comparison between the adult female population in treated and untreated plants (total population needles + twigs), the effect of endotherapy was underlined by differences in population density over the time. In fact, during this period, while the population size on untreated plants reported a peak of population density, the population in treated plants maintained a low level until the first half of September. We may therefore conclude that the effect of the treatment observed in the population trends is proportionally related with the content of abamectin in plant tissues provided by the multiresidue analysis. A noticeable reduction of the population recorded on treated plants occurred approximately 45 days after the treatment. Given this result, it follows that the effect of the treatment is not immediate. A likely explanation of this delay is the time required by pine plants to absorb and spread abamectin among organs and tissues^23^ and it is an aspect worthy of exploration in future studies. Different causes may be responsible for the timing of abamectin effectiveness. As reported in existing literature, the spread of active ingredients into plant tissues after endotherapy may be caused by the anatomy and physiology of the plant, to the climatic conditions of the living environment and to the characteristics of the agrochemicals.^23^, ^24^

Few studies, in the existing literature, deeply explored the pest population abundance and the concentration of abamectin within plants,^25^, ^26^, ^27^ even fewer have carried out a residue analysis of the active ingredients.^14^, ^28^, ^29^ To the best of our knowledge, only Dembilio *et al*.^14^ have carried out a very similar experiment on palms, a species with different physiological mechanisms in respect of *P. pinea*. Their results show a timing of absorption and diffusion and a maximum concentration of abamectin in line with our results.

From the comparison between the trends of the population on treated plants and of the abamectin concentration it seems that the population starts to significantly decrease when the maximum peak of abamectin occurs. Future studies, however, should be extended to explore the effects of abamectin treatments on the other insects’ life stages besides adult females, in order to individuate the most susceptible one.

Concerning pest management, an additional and relevant finding is the persistence of abamectin into plants. We found that it is limited to approximately 2 months, after which we observed a slow increase in population density on treated plants. In fact, as stated in other scientific works^14^, ^30^ it was of great importance to understand how long abamectin persists inside the plant vessels. Our results are not in accordance with Dembilio *et al*.,^14^ since they reported a longer persistence (up to 5 months) on palms. Having a limited persistence of the active ingredient into plants could have a relevant impact on planning the control of *Toumeyella parvicornis* for two main reasons: (i) how many treatments are necessary during the year to maintain the pest population level below a critical threshold should be investigated, and (ii) the effect that multiple endotherapic treatments carried out in a single year may have on plant health should be explored further.^31^, ^32^

Otherwise, there is a potential advantage in giving abamectin through endotherapy. Stone pine plants are one of the most diffused species in urban and suburban areas of central Italy and in many areas of southwest Europe.^33^ Given the environment and high‐density population in cities, spraying active ingredients is not allowed by National law.^34^ Accordingly, when high infestations occur, control actions are strongly limited and, if other attempts cannot be implemented, the plants are often removed. Endotherapy in this context may be a compromise to ensure an efficacy control of the pest populations and a right safeguard of the environment.

## CONFLICT OF INTEREST

The authors declare that they have no conflict of interest.

## AUTHOR CONTRIBUTIONS

NDS, LR, MC and SS conceived research. NDS, LR and MC wrote the manuscript. NDS and EC conducted the experiments. NDS and LR conducted the statistical analyses. SS secured fundings. All the authors read, contributed to and approved the manuscript.

## Data Availability

The data associated with this publication, as well as the R script to reproduce the results, are publicly available at https://github.com/lucaros1190/Toumpa-dataset.
